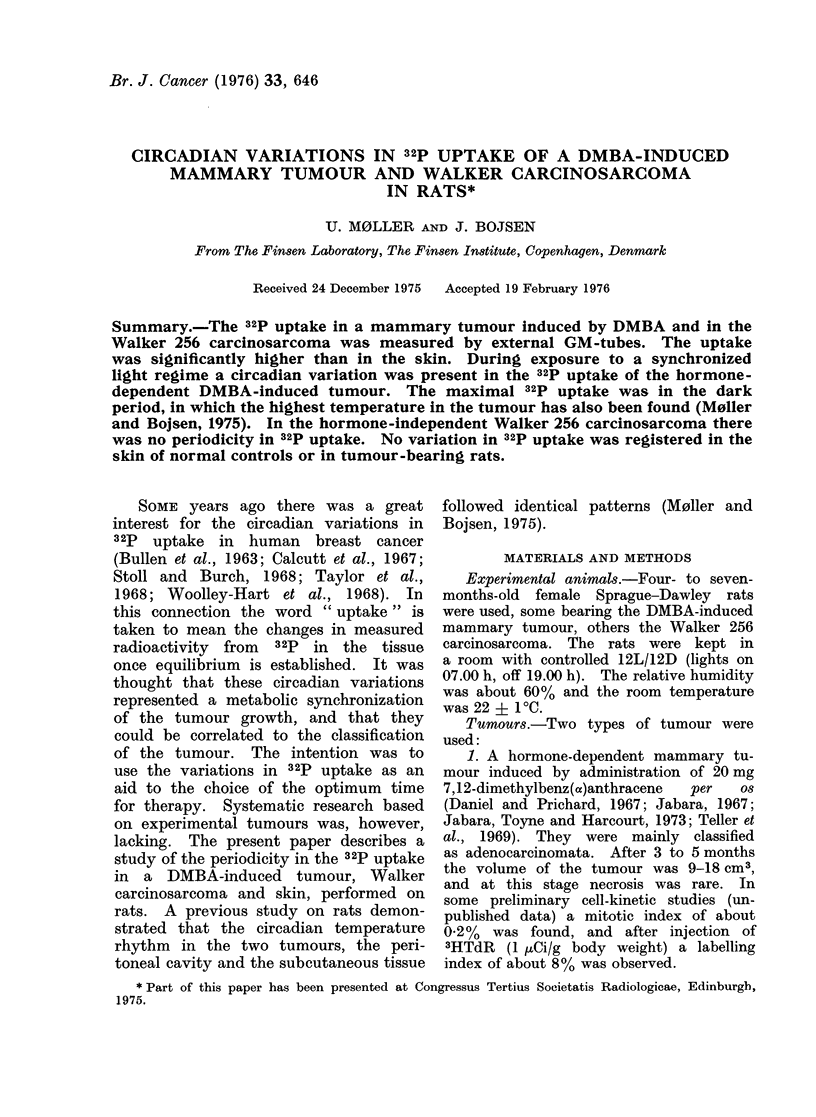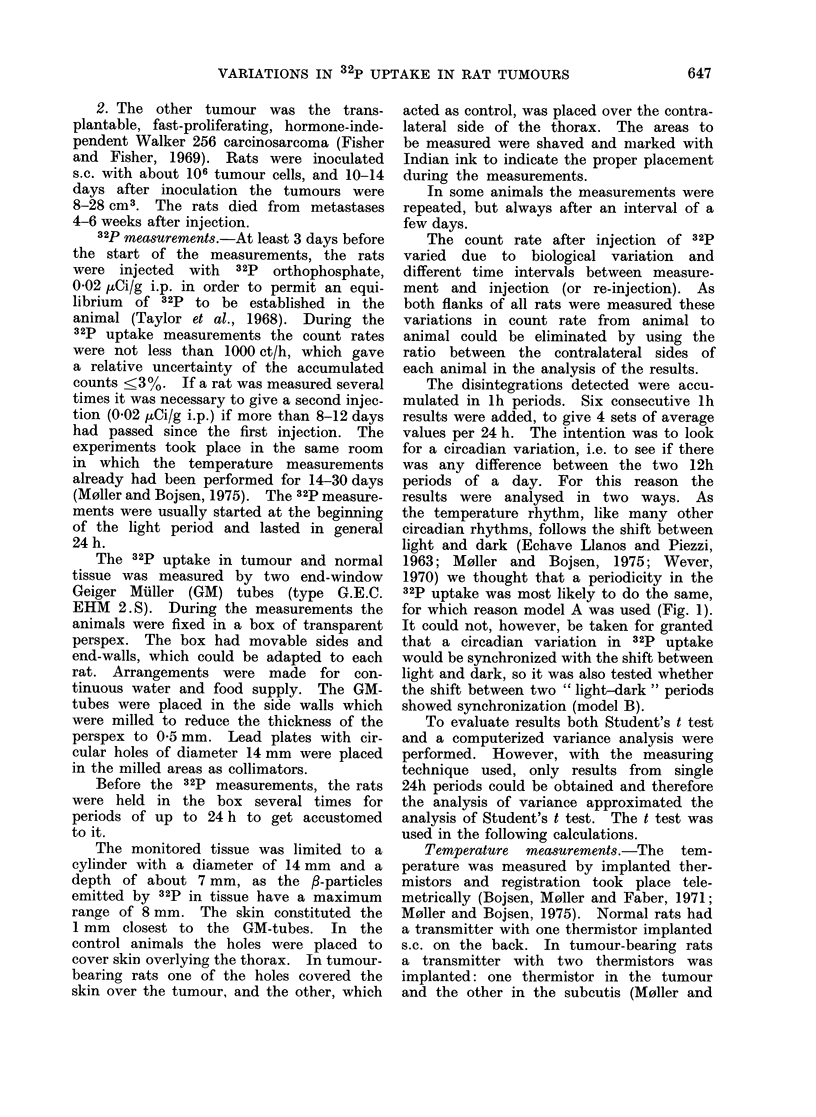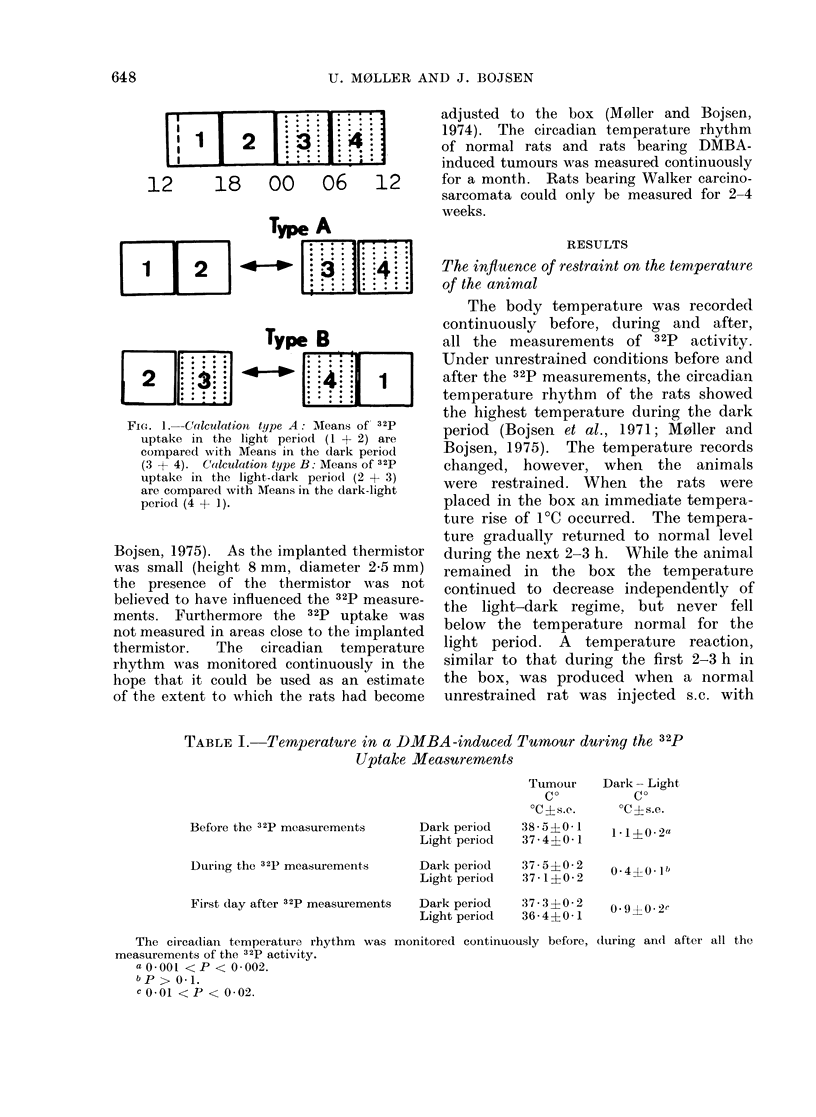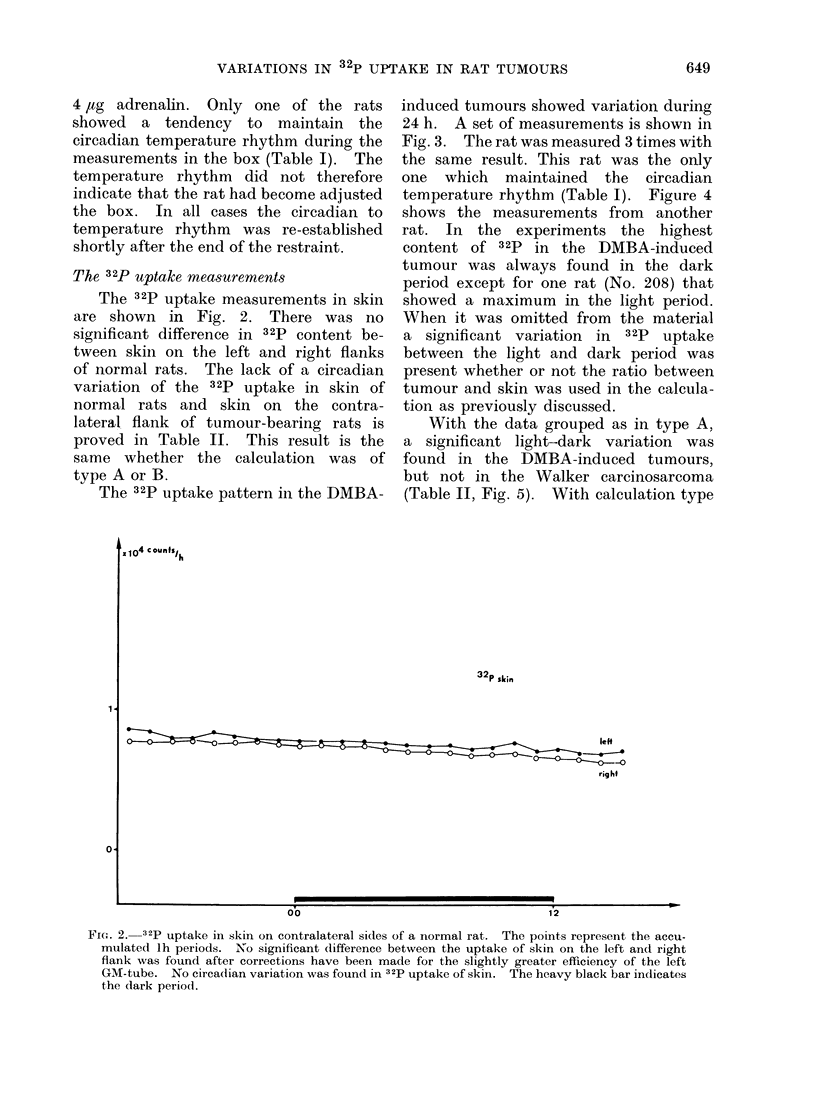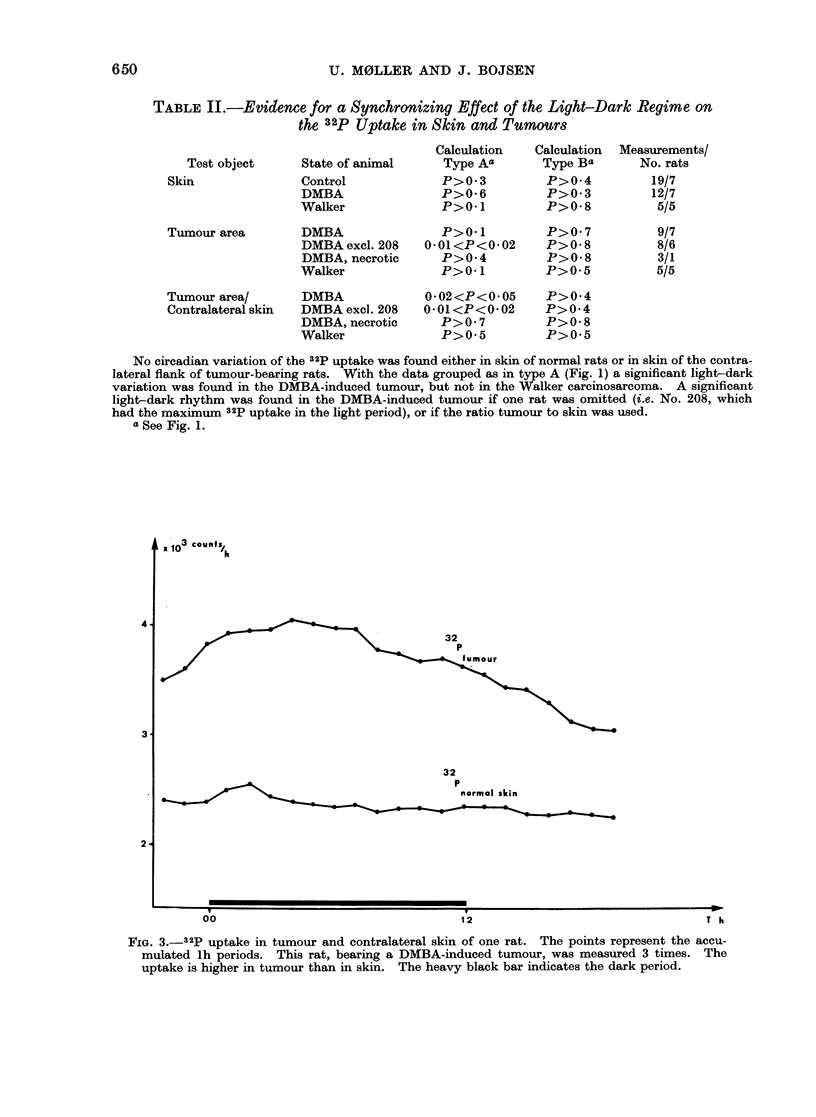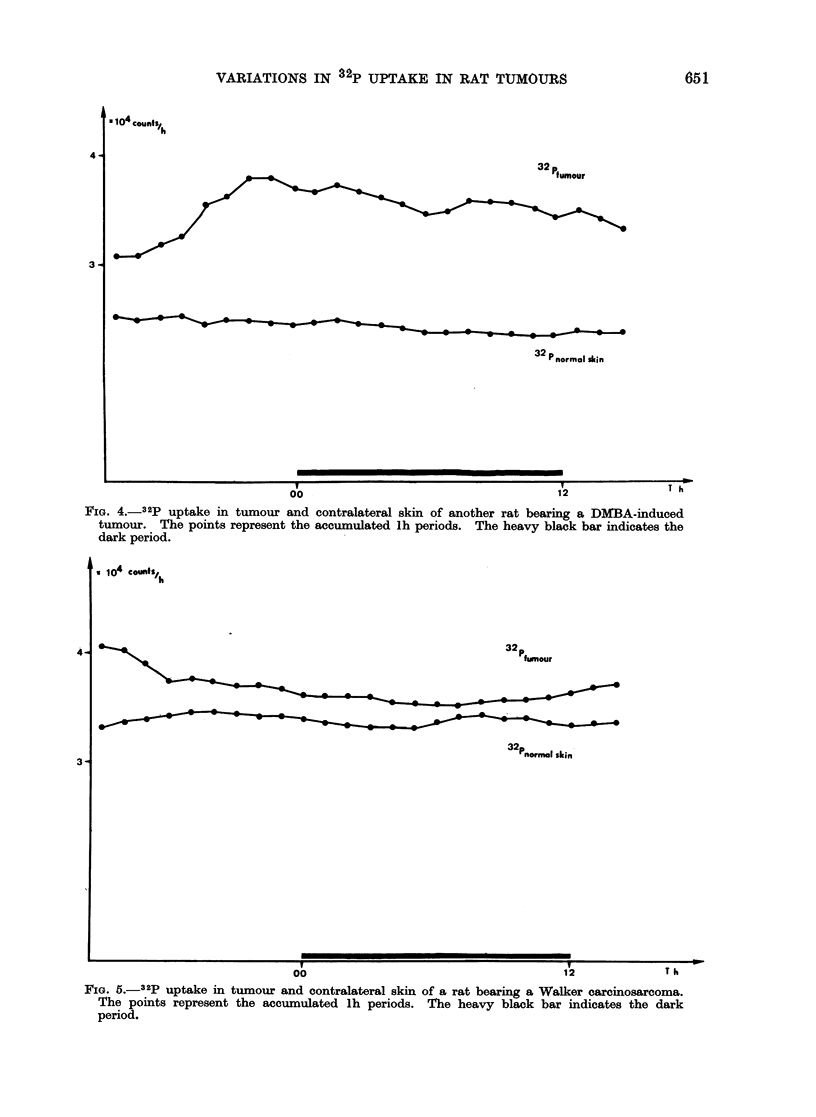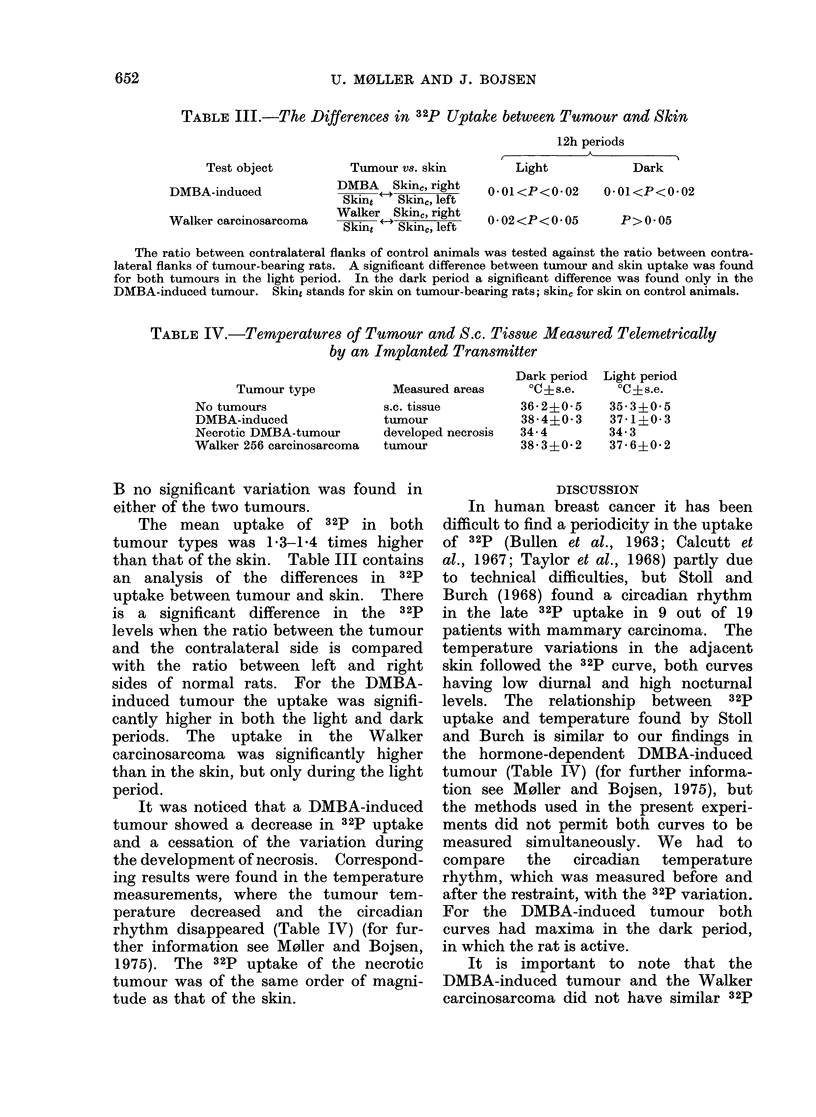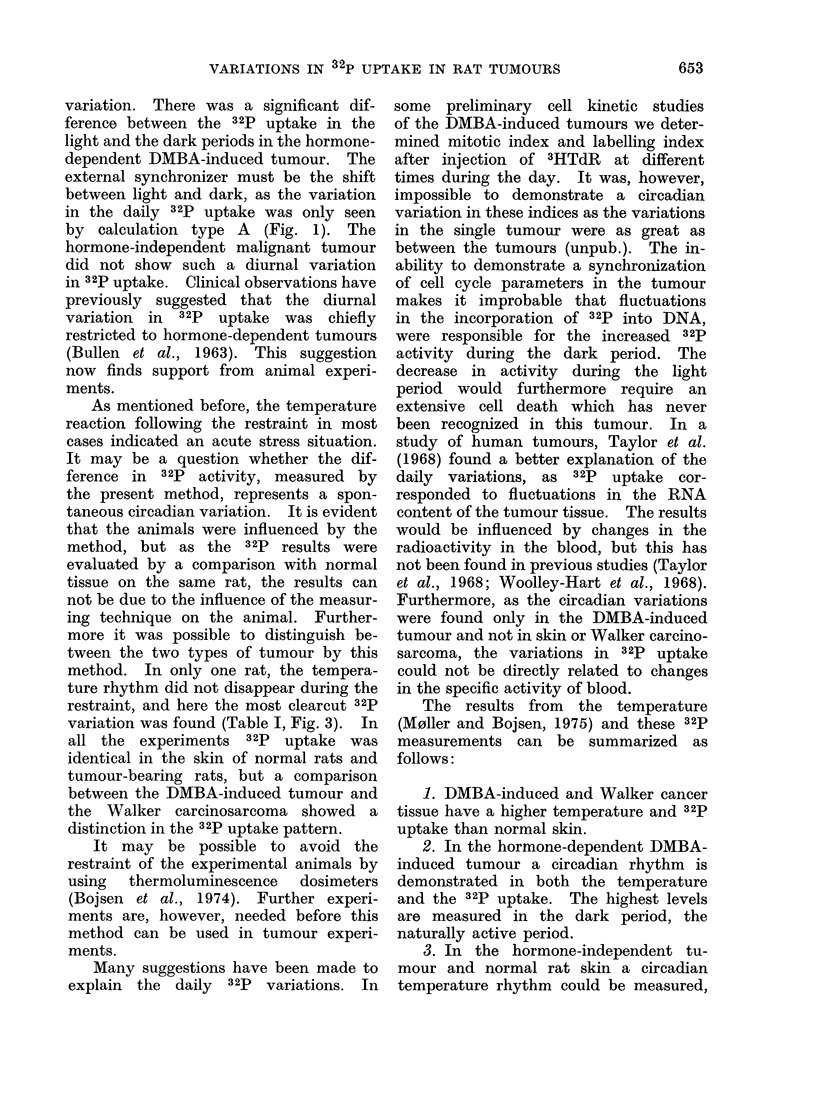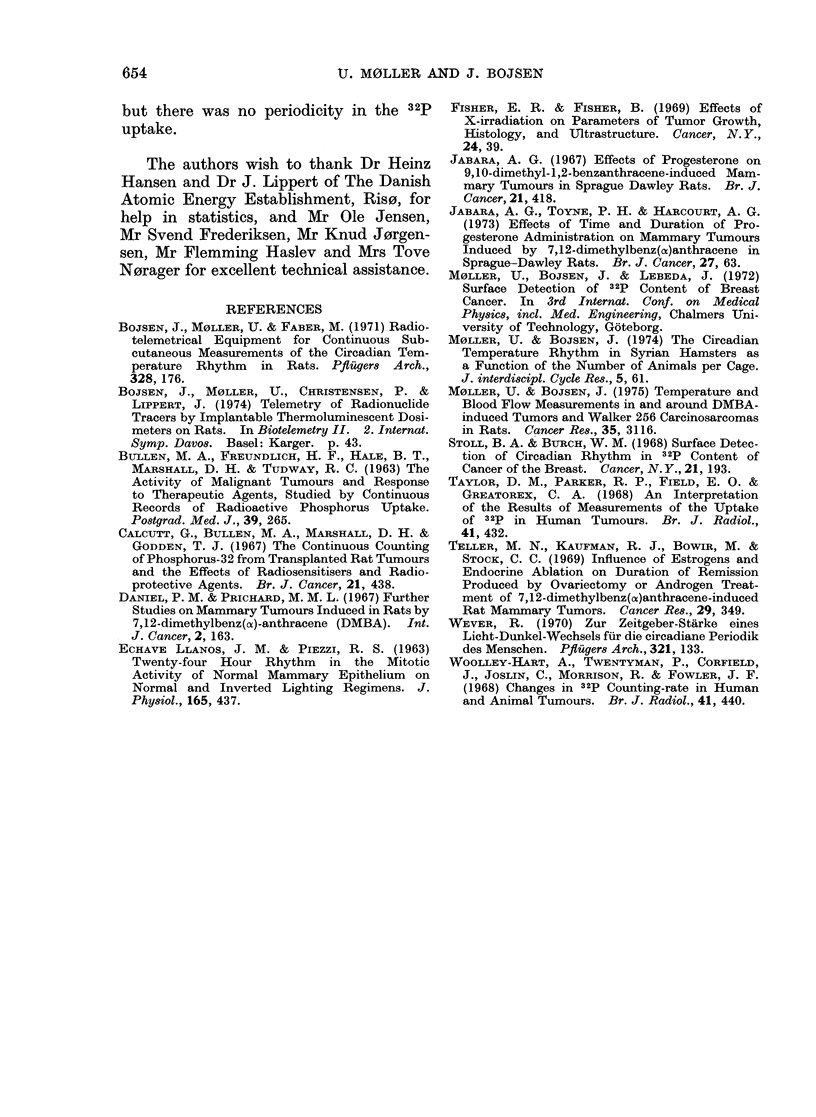# Circadian variations in 32P uptake of DMBA-induced mammary tumour and Walker carcinosarcoma in rats.

**DOI:** 10.1038/bjc.1976.102

**Published:** 1976-06

**Authors:** U. Møoller, J. Bojsen

## Abstract

The 32P uptake in a mammary tumour induced by DMBA and in the Walker 256 carcinosarcoma was measured by external GM -tubes. The uptake was significantly higher than in the skin. During exposure to a synchronized light regime a circadian variation was present in the 32P uptake of the hormone-dependent DMBA-induced tumour. The maximal 32P uptake was in the dark period, in which the highest temperature in the tumour has also been found (Møoller and Bojsen, 1975). In the hormone-independent Walker 256 carcinosarcoma there was no periodicity in 32P uptake. No variation in 32P uptake was registered in the skin of normal controls or in tumour-bearing rats.


*Br. J. Cancer* (1976) **33**, 646

### CIRCADIAN VARIATIONS IN $^{32}P$ UPTAKE OF A DMBA-INDUCED MAMMARY TUMOUR AND WALKER CARCINOSARCOMA IN RATS*

U. MØLLER AND J. BOJSEN

*From The Finsen Laboratory, The Finsen Institute, Copenhagen, Denmark*

Received 24 December 1975 Accepted 19 February 1976

**Summary.—The $^{32}P$ uptake in a mammary tumour induced by DMBA and in the Walker 256 carcinosarcoma was measured by external GM-tubes. The uptake was significantly higher than in the skin. During exposure to a synchronized light regime a circadian variation was present in the $^{32}P$ uptake of the hormone-dependent DMBA-induced tumour. The maximal $^{32}P$ uptake was in the dark period, in which the highest temperature in the tumour has also been found (Møller and Bojsen, 1975). In the hormone-independent Walker 256 carcinosarcoma there was no periodicity in $^{32}P$ uptake. No variation in $^{32}P$ uptake was registered in the skin of normal controls or in tumour-bearing rats.**

SOME years ago there was a great interest for the circadian variations in $^{32}P$ uptake in human breast cancer (Bullen *et al.*, 1963; Calcutt *et al.*, 1967; Stoll and Burch, 1968; Taylor *et al.*, 1968; Woolley-Hart *et al.*, 1968). In this connection the word " uptake " is taken to mean the changes in measured radioactivity from $^{32}P$ in the tissue once equilibrium is established. It was thought that these circadian variations represented a metabolic synchronization of the tumour growth, and that they could be correlated to the classification of the tumour. The intention was to use the variations in $^{32}P$ uptake as an aid to the choice of the optimum time for therapy. Systematic research based on experimental tumours was, however, lacking. The present paper describes a study of the periodicity in the $^{32}P$ uptake in a DMBA-induced tumour, Walker carcinosarcoma and skin, performed on rats. A previous study on rats demonstrated that the circadian temperature rhythm in the two tumours, the peritoneal cavity and the subcutaneous tissue followed identical patterns (Møller and Bojsen, 1975).

### MATERIALS AND METHODS

*Experimental animals.*—Four- to seven-months-old female Sprague–Dawley rats were used, some bearing the DMBA-induced mammary tumour, others the Walker 256 carcinosarcoma. The rats were kept in a room with controlled 12L/12D (lights on 07.00 h, off 19.00 h). The relative humidity was about 60% and the room temperature was 22 ± 1°C.

*Tumours.*—Two types of tumour were used:

*1.* A hormone-dependent mammary tumour induced by administration of 20 mg 7,12-dimethylbenz(α)anthracene *per os* (Daniel and Prichard, 1967; Jabara, 1967; Jabara, Toyne and Harcourt, 1973; Teller *et al.*, 1969). They were mainly classified as adenocarcinomata. After 3 to 5 months the volume of the tumour was 9–18 cm$^3$, and at this stage necrosis was rare. In some preliminary cell-kinetic studies (unpublished data) a mitotic index of about 0·2% was found, and after injection of $^{3}$HTdR (1 μCi/g body weight) a labelling index of about 8% was observed.

* Part of this paper has been presented at Congressus Tertius Societatis Radiologicae, Edinburgh, 1975.

*2.* The other tumour was the transplantable, fast-proliferating, hormone-independent Walker 256 carcinosarcoma (Fisher and Fisher, 1969). Rats were inoculated s.c. with about $10^6$ tumour cells, and 10–14 days after inoculation the tumours were 8–28 $cm^3$. The rats died from metastases 4–6 weeks after injection.

*$^{32}P$ measurements.*—At least 3 days before the start of the measurements, the rats were injected with $^{32}P$ orthophosphate, 0·02 $\mu$Ci/g i.p. in order to permit an equilibrium of $^{32}P$ to be established in the animal (Taylor *et al.*, 1968). During the $^{32}P$ uptake measurements the count rates were not less than 1000 ct/h, which gave a relative uncertainty of the accumulated counts $\leq 3\%$. If a rat was measured several times it was necessary to give a second injection (0·02 $\mu$Ci/g i.p.) if more than 8–12 days had passed since the first injection. The experiments took place in the same room in which the temperature measurements already had been performed for 14–30 days (Møller and Bojsen, 1975). The $^{32}P$ measurements were usually started at the beginning of the light period and lasted in general 24 h.

The $^{32}P$ uptake in tumour and normal tissue was measured by two end-window Geiger Müller (GM) tubes (type G.E.C. EHM 2.S). During the measurements the animals were fixed in a box of transparent perspex. The box had movable sides and end-walls, which could be adapted to each rat. Arrangements were made for continuous water and food supply. The GM-tubes were placed in the side walls which were milled to reduce the thickness of the perspex to 0·5 mm. Lead plates with circular holes of diameter 14 mm were placed in the milled areas as collimators.

Before the $^{32}P$ measurements, the rats were held in the box several times for periods of up to 24 h to get accustomed to it.

The monitored tissue was limited to a cylinder with a diameter of 14 mm and a depth of about 7 mm, as the $\beta$-particles emitted by $^{32}P$ in tissue have a maximum range of 8 mm. The skin constituted the 1 mm closest to the GM-tubes. In the control animals the holes were placed to cover skin overlying the thorax. In tumour-bearing rats one of the holes covered the skin over the tumour, and the other, which acted as control, was placed over the contralateral side of the thorax. The areas to be measured were shaved and marked with Indian ink to indicate the proper placement during the measurements.

In some animals the measurements were repeated, but always after an interval of a few days.

The count rate after injection of $^{32}P$ varied due to biological variation and different time intervals between measurement and injection (or re-injection). As both flanks of all rats were measured these variations in count rate from animal to animal could be eliminated by using the ratio between the contralateral sides of each animal in the analysis of the results.

The disintegrations detected were accumulated in 1h periods. Six consecutive 1h results were added, to give 4 sets of average values per 24 h. The intention was to look for a circadian variation, i.e. to see if there was any difference between the two 12h periods of a day. For this reason the results were analysed in two ways. As the temperature rhythm, like many other circadian rhythms, follows the shift between light and dark (Echave Llanos and Piezzi, 1963; Møller and Bojsen, 1975; Wever, 1970) we thought that a periodicity in the $^{32}P$ uptake was most likely to do the same, for which reason model A was used (Fig. 1). It could not, however, be taken for granted that a circadian variation in $^{32}P$ uptake would be synchronized with the shift between light and dark, so it was also tested whether the shift between two " light–dark " periods showed synchronization (model B).

To evaluate results both Student's *t* test and a computerized variance analysis were performed. However, with the measuring technique used, only results from single 24h periods could be obtained and therefore the analysis of variance approximated the analysis of Student's *t* test. The *t* test was used in the following calculations.

*Temperature measurements.*—The temperature was measured by implanted thermistors and registration took place telemetrically (Bojsen, Møller and Faber, 1971; Møller and Bojsen, 1975). Normal rats had a transmitter with one thermistor implanted s.c. on the back. In tumour-bearing rats a transmitter with two thermistors was implanted: one thermistor in the tumour and the other in the subcutis (Møller and

FIG. 1.—*Calculation type A:* Means of $^{32}P$ uptake in the light period (1 + 2) are compared with Means in the dark period (3 + 4). *Calculation type B:* Means of $^{32}P$ uptake in the light-dark period (2 + 3) are compared with Means in the dark-light period (4 + 1).

Bojsen, 1975). As the implanted thermistor was small (height 8 mm, diameter 2·5 mm) the presence of the thermistor was not believed to have influenced the $^{32}P$ measurements. Furthermore the $^{32}P$ uptake was not measured in areas close to the implanted thermistor. The circadian temperature rhythm was monitored continuously in the hope that it could be used as an estimate of the extent to which the rats had become adjusted to the box (Møller and Bojsen, 1974). The circadian temperature rhythm of normal rats and rats bearing DMBA-induced tumours was measured continuously for a month. Rats bearing Walker carcinosarcomata could only be measured for 2–4 weeks.

### RESULTS

### *The influence of restraint on the temperature of the animal*

The body temperature was recorded continuously before, during and after, all the measurements of $^{32}P$ activity. Under unrestrained conditions before and after the $^{32}P$ measurements, the circadian temperature rhythm of the rats showed the highest temperature during the dark period (Bojsen *et al.*, 1971; Møller and Bojsen, 1975). The temperature records changed, however, when the animals were restrained. When the rats were placed in the box an immediate temperature rise of 1°C occurred. The temperature gradually returned to normal level during the next 2–3 h. While the animal remained in the box the temperature continued to decrease independently of the light–dark regime, but never fell below the temperature normal for the light period. A temperature reaction, similar to that during the first 2–3 h in the box, was produced when a normal unrestrained rat was injected s.c. with

TABLE I.—*Temperature in a DMBA-induced Tumour during the $^{32}P$ Uptake Measurements*

| | | Tumour C° °C±s.e. | Dark – Light C° °C±s.e. |
|---|---|---|---|
| Before the $^{32}P$ measurements | Dark period | 38·5±0·1 | 1·1±0·2[a] |
| | Light period | 37·4±0·1 | |
| During the $^{32}P$ measurements | Dark period | 37·5±0·2 | 0·4±0·1[b] |
| | Light period | 37·1±0·2 | |
| First day after $^{32}P$ measurements | Dark period | 37·3±0·2 | 0·9±0·2[c] |
| | Light period | 36·4±0·1 | |

The circadian temperature rhythm was monitored continuously before, during and after all the measurements of the $^{32}P$ activity.

[a] $0·001 < P < 0·002$.

[b] $P > 0·1$.

[c] $0·01 < P < 0·02$.

4 $\mu$g adrenalin. Only one of the rats showed a tendency to maintain the circadian temperature rhythm during the measurements in the box (Table I). The temperature rhythm did not therefore indicate that the rat had become adjusted the box. In all cases the circadian to temperature rhythm was re-established shortly after the end of the restraint.

*The $^{32}P$ uptake measurements*

The $^{32}$P uptake measurements in skin are shown in Fig. 2. There was no significant difference in $^{32}$P content between skin on the left and right flanks of normal rats. The lack of a circadian variation of the $^{32}$P uptake in skin of normal rats and skin on the contralateral flank of tumour-bearing rats is proved in Table II. This result is the same whether the calculation was of type A or B.

The $^{32}$P uptake pattern in the DMBA-induced tumours showed variation during 24 h. A set of measurements is shown in Fig. 3. The rat was measured 3 times with the same result. This rat was the only one which maintained the circadian temperature rhythm (Table I). Figure 4 shows the measurements from another rat. In the experiments the highest content of $^{32}$P in the DMBA-induced tumour was always found in the dark period except for one rat (No. 208) that showed a maximum in the light period. When it was omitted from the material a significant variation in $^{32}$P uptake between the light and dark period was present whether or not the ratio between tumour and skin was used in the calculation as previously discussed.

With the data grouped as in type A, a significant light–dark variation was found in the DMBA-induced tumours, but not in the Walker carcinosarcoma (Table II, Fig. 5). With calculation type

FIG. 2.—$^{32}$P uptake in skin on contralateral sides of a normal rat. The points represent the accumulated 1h periods. No significant difference between the uptake of skin on the left and right flank was found after corrections have been made for the slightly greater efficiency of the left GM-tube. No circadian variation was found in $^{32}$P uptake of skin. The heavy black bar indicates the dark period.

TABLE II.—*Evidence for a Synchronizing Effect of the Light–Dark Regime on the $^{32}P$ Uptake in Skin and Tumours*

| Test object | State of animal | Calculation Type A[a] | Calculation Type B[a] | Measurements/ No. rats |
|---|---|---|---|---|
| Skin | Control | $P>0\cdot3$ | $P>0\cdot4$ | 19/7 |
| | DMBA | $P>0\cdot6$ | $P>0\cdot3$ | 12/7 |
| | Walker | $P>0\cdot1$ | $P>0\cdot8$ | 5/5 |
| Tumour area | DMBA | $P>0\cdot1$ | $P>0\cdot7$ | 9/7 |
| | DMBA excl. 208 | $0\cdot01<P<0\cdot02$ | $P>0\cdot8$ | 8/6 |
| | DMBA, necrotic | $P>0\cdot4$ | $P>0\cdot8$ | 3/1 |
| | Walker | $P>0\cdot1$ | $P>0\cdot5$ | 5/5 |
| Tumour area/ Contralateral skin | DMBA | $0\cdot02<P<0\cdot05$ | $P>0\cdot4$ | |
| | DMBA excl. 208 | $0\cdot01<P<0\cdot02$ | $P>0\cdot4$ | |
| | DMBA, necrotic | $P>0\cdot7$ | $P>0\cdot8$ | |
| | Walker | $P>0\cdot5$ | $P>0\cdot5$ | |

No circadian variation of the $^{32}P$ uptake was found either in skin of normal rats or in skin of the contralateral flank of tumour-bearing rats. With the data grouped as in type A (Fig. 1) a significant light–dark variation was found in the DMBA-induced tumour, but not in the Walker carcinosarcoma. A significant light–dark rhythm was found in the DMBA-induced tumour if one rat was omitted (*i.e.* No. 208, which had the maximum $^{32}P$ uptake in the light period), or if the ratio tumour to skin was used.

[a] See Fig. 1.

FIG. 3.—$^{32}P$ uptake in tumour and contralateral skin of one rat. The points represent the accumulated 1h periods. This rat, bearing a DMBA-induced tumour, was measured 3 times. The uptake is higher in tumour than in skin. The heavy black bar indicates the dark period.

FIG. 4.—$^{32}$P uptake in tumour and contralateral skin of another rat bearing a DMBA-induced tumour. The points represent the accumulated 1h periods. The heavy black bar indicates the dark period.

FIG. 5.—$^{32}$P uptake in tumour and contralateral skin of a rat bearing a Walker carcinosarcoma. The points represent the accumulated 1h periods. The heavy black bar indicates the dark period.

TABLE III.—*The Differences in $^{32}P$ Uptake between Tumour and Skin*

| Test object | Tumour *vs.* skin | 12h periods: Light | Dark |
|---|---|---|---|
| DMBA-induced | $\frac{DMBA}{Skin_t} \leftrightarrow \frac{Skin_c, right}{Skin_c, left}$ | $0 \cdot 01 < P < 0 \cdot 02$ | $0 \cdot 01 < P < 0 \cdot 02$ |
| Walker carcinosarcoma | $\frac{Walker}{Skin_t} \leftrightarrow \frac{Skin_c, right}{Skin_c, left}$ | $0 \cdot 02 < P < 0 \cdot 05$ | $P > 0 \cdot 05$ |

The ratio between contralateral flanks of control animals was tested against the ratio between contralateral flanks of tumour-bearing rats. A significant difference between tumour and skin uptake was found for both tumours in the light period. In the dark period a significant difference was found only in the DMBA-induced tumour. $Skin_t$ stands for skin on tumour-bearing rats; $skin_c$ for skin on control animals.

TABLE IV.—*Temperatures of Tumour and S.c. Tissue Measured Telemetrically by an Implanted Transmitter*

| Tumour type | Measured areas | Dark period °C±s.e. | Light period °C±s.e. |
|---|---|---|---|
| No tumours | s.c. tissue | 36·2±0·5 | 35·3±0·5 |
| DMBA-induced | tumour | 38·4±0·3 | 37·1±0·3 |
| Necrotic DMBA-tumour | developed necrosis | 34·4 | 34·3 |
| Walker 256 carcinosarcoma | tumour | 38·3±0·2 | 37·6±0·2 |

B no significant variation was found in either of the two tumours.

The mean uptake of $^{32}P$ in both tumour types was 1·3–1·4 times higher than that of the skin. Table III contains an analysis of the differences in $^{32}P$ uptake between tumour and skin. There is a significant difference in the $^{32}P$ levels when the ratio between the tumour and the contralateral side is compared with the ratio between left and right sides of normal rats. For the DMBA-induced tumour the uptake was significantly higher in both the light and dark periods. The uptake in the Walker carcinosarcoma was significantly higher than in the skin, but only during the light period.

It was noticed that a DMBA-induced tumour showed a decrease in $^{32}P$ uptake and a cessation of the variation during the development of necrosis. Corresponding results were found in the temperature measurements, where the tumour temperature decreased and the circadian rhythm disappeared (Table IV) (for further information see Møller and Bojsen, 1975). The $^{32}P$ uptake of the necrotic tumour was of the same order of magnitude as that of the skin.

### DISCUSSION

In human breast cancer it has been difficult to find a periodicity in the uptake of $^{32}P$ (Bullen *et al.*, 1963; Calcutt *et al.*, 1967; Taylor *et al.*, 1968) partly due to technical difficulties, but Stoll and Burch (1968) found a circadian rhythm in the late $^{32}P$ uptake in 9 out of 19 patients with mammary carcinoma. The temperature variations in the adjacent skin followed the $^{32}P$ curve, both curves having low diurnal and high nocturnal levels. The relationship between $^{32}P$ uptake and temperature found by Stoll and Burch is similar to our findings in the hormone-dependent DMBA-induced tumour (Table IV) (for further information see Møller and Bojsen, 1975), but the methods used in the present experiments did not permit both curves to be measured simultaneously. We had to compare the circadian temperature rhythm, which was measured before and after the restraint, with the $^{32}P$ variation. For the DMBA-induced tumour both curves had maxima in the dark period, in which the rat is active.

It is important to note that the DMBA-induced tumour and the Walker carcinosarcoma did not have similar $^{32}P$

variation. There was a significant difference between the $^{32}P$ uptake in the light and the dark periods in the hormone-dependent DMBA-induced tumour. The external synchronizer must be the shift between light and dark, as the variation in the daily $^{32}P$ uptake was only seen by calculation type A (Fig. 1). The hormone-independent malignant tumour did not show such a diurnal variation in $^{32}P$ uptake. Clinical observations have previously suggested that the diurnal variation in $^{32}P$ uptake was chiefly restricted to hormone-dependent tumours (Bullen *et al.*, 1963). This suggestion now finds support from animal experiments.

As mentioned before, the temperature reaction following the restraint in most cases indicated an acute stress situation. It may be a question whether the difference in $^{32}P$ activity, measured by the present method, represents a spontaneous circadian variation. It is evident that the animals were influenced by the method, but as the $^{32}P$ results were evaluated by a comparison with normal tissue on the same rat, the results can not be due to the influence of the measuring technique on the animal. Furthermore it was possible to distinguish between the two types of tumour by this method. In only one rat, the temperature rhythm did not disappear during the restraint, and here the most clearcut $^{32}P$ variation was found (Table I, Fig. 3). In all the experiments $^{32}P$ uptake was identical in the skin of normal rats and tumour-bearing rats, but a comparison between the DMBA-induced tumour and the Walker carcinosarcoma showed a distinction in the $^{32}P$ uptake pattern.

It may be possible to avoid the restraint of the experimental animals by using thermoluminescence dosimeters (Bojsen *et al.*, 1974). Further experiments are, however, needed before this method can be used in tumour experiments.

Many suggestions have been made to explain the daily $^{32}P$ variations. In some preliminary cell kinetic studies of the DMBA-induced tumours we determined mitotic index and labelling index after injection of $^{3}$HTdR at different times during the day. It was, however, impossible to demonstrate a circadian variation in these indices as the variations in the single tumour were as great as between the tumours (unpub.). The inability to demonstrate a synchronization of cell cycle parameters in the tumour makes it improbable that fluctuations in the incorporation of $^{32}P$ into DNA, were responsible for the increased $^{32}P$ activity during the dark period. The decrease in activity during the light period would furthermore require an extensive cell death which has never been recognized in this tumour. In a study of human tumours, Taylor *et al.* (1968) found a better explanation of the daily variations, as $^{32}P$ uptake corresponded to fluctuations in the RNA content of the tumour tissue. The results would be influenced by changes in the radioactivity in the blood, but this has not been found in previous studies (Taylor *et al.*, 1968; Woolley-Hart *et al.*, 1968). Furthermore, as the circadian variations were found only in the DMBA-induced tumour and not in skin or Walker carcinosarcoma, the variations in $^{32}P$ uptake could not be directly related to changes in the specific activity of blood.

The results from the temperature (Møller and Bojsen, 1975) and these $^{32}P$ measurements can be summarized as follows:

*1.* DMBA-induced and Walker cancer tissue have a higher temperature and $^{32}P$ uptake than normal skin.

*2.* In the hormone-dependent DMBA-induced tumour a circadian rhythm is demonstrated in both the temperature and the $^{32}P$ uptake. The highest levels are measured in the dark period, the naturally active period.

*3.* In the hormone-independent tumour and normal rat skin a circadian temperature rhythm could be measured, but there was no periodicity in the $^{32}P$ uptake.

The authors wish to thank Dr Heinz Hansen and Dr J. Lippert of The Danish Atomic Energy Establishment, Risø, for help in statistics, and Mr Ole Jensen, Mr Svend Frederiksen, Mr Knud Jørgensen, Mr Flemming Haslev and Mrs Tove Nørager for excellent technical assistance.

### REFERENCES

BOJSEN, J., MØLLER, U. & FABER, M. (1971) Radiotelemetrical Equipment for Continuous Subcutaneous Measurements of the Circadian Temperature Rhythm in Rats. *Pflügers Arch.*, **328**, 176.

BOJSEN, J., MØLLER, U., CHRISTENSEN, P. & LIPPERT, J. (1974) Telemetry of Radionuclide Tracers by Implantable Thermoluminescent Dosimeters on Rats. In *Biotelemetry II. 2. Internat. Symp. Davos.* Basel: Karger. p. 43.

BULLEN, M. A., FREUNDLICH, H. F., HALE, B. T., MARSHALL, D. H. & TUDWAY, R. C. (1963) The Activity of Malignant Tumours and Response to Therapeutic Agents, Studied by Continuous Records of Radioactive Phosphorus Uptake. *Postgrad. Med. J.*, **39**, 265.

CALCUTT, G., BULLEN, M. A., MARSHALL, D. H. & GODDEN, T. J. (1967) The Continuous Counting of Phosphorus-32 from Transplanted Rat Tumours and the Effects of Radiosensitisers and Radioprotective Agents. *Br. J. Cancer*, **21**, 438.

DANIEL, P. M. & PRICHARD, M. M. L. (1967) Further Studies on Mammary Tumours Induced in Rats by 7,12-dimethylbenz(α)-anthracene (DMBA). *Int. J. Cancer*, **2**, 163.

ECHAVE LLANOS, J. M. & PIEZZI, R. S. (1963) Twenty-four Hour Rhythm in the Mitotic Activity of Normal Mammary Epithelium on Normal and Inverted Lighting Regimens. *J. Physiol.*, **165**, 437.

FISHER, E. R. & FISHER, B. (1969) Effects of X-irradiation on Parameters of Tumor Growth, Histology, and Ultrastructure. *Cancer, N.Y.*, **24**, 39.

JABARA, A. G. (1967) Effects of Progesterone on 9,10-dimethyl-1,2-benzanthracene-induced Mammary Tumours in Sprague Dawley Rats. *Br. J. Cancer*, **21**, 418.

JABARA, A. G., TOYNE, P. H. & HARCOURT, A. G. (1973) Effects of Time and Duration of Progesterone Administration on Mammary Tumours Induced by 7,12-dimethylbenz(α)anthracene in Sprague–Dawley Rats. *Br. J. Cancer*, **27**, 63.

MØLLER, U., BOJSEN, J. & LEBEDA, J. (1972) Surface Detection of $^{32}P$ Content of Breast Cancer. In *3rd Internat. Conf. on Medical Physics, incl. Med. Engineering*, Chalmers University of Technology, Göteborg.

MØLLER, U. & BOJSEN, J. (1974) The Circadian Temperature Rhythm in Syrian Hamsters as a Function of the Number of Animals per Cage. *J. interdiscipl. Cycle Res.*, **5**, 61.

MØLLER, U. & BOJSEN, J. (1975) Temperature and Blood Flow Measurements in and around DMBA-induced Tumors and Walker 256 Carcinosarcomas in Rats. *Cancer Res.*, **35**, 3116.

STOLL, B. A. & BURCH, W. M. (1968) Surface Detection of Circadian Rhythm in $^{32}P$ Content of Cancer of the Breast. *Cancer, N.Y.*, **21**, 193.

TAYLOR, D. M., PARKER, R. P., FIELD, E. O. & GREATOREX, C. A. (1968) An Interpretation of the Results of Measurements of the Uptake of $^{32}P$ in Human Tumours. *Br. J. Radiol.*, **41**, 432.

TELLER, M. N., KAUFMAN, R. J., BOWIR, M. & STOCK, C. C. (1969) Influence of Estrogens and Endocrine Ablation on Duration of Remission Produced by Ovariectomy or Androgen Treatment of 7,12-dimethylbenz(α)anthracene-induced Rat Mammary Tumors. *Cancer Res.*, **29**, 349.

WEVER, R. (1970) Zur Zeitgeber-Stärke eines Licht-Dunkel-Wechsels für die circadiane Periodik des Menschen. *Pflügers Arch.*, **321**, 133.

WOOLLEY-HART, A., TWENTYMAN, P., CORFIELD, J., JOSLIN, C., MORRISON, R. & FOWLER, J. F. (1968) Changes in $^{32}P$ Counting-rate in Human and Animal Tumours. *Br. J. Radiol.*, **41**, 440.